# Mental health status of informal waste workers during the COVID-19 pandemic in Bangladesh

**DOI:** 10.1371/journal.pone.0262141

**Published:** 2022-01-07

**Authors:** Md. Rajwanul Haque, Md. Mostaured Ali Khan, Md. Mosfequr Rahman, M. Sajjadur Rahman, Shawkat A. Begum

**Affiliations:** 1 MEL and Research, Practical Action, Dhanmondi, Dhaka, Bangladesh; 2 Department of Population Science and Human Resource Development, University of Rajshahi, Rajshahi, Bangladesh; 3 Practical Action, Dhanmondi, Dhaka, Bangladesh; National Institutes of Health, UNITED STATES

## Abstract

The deadliest coronavirus disease 2019 (COVID-19) is taking thousands of lives worldwide and presents an extraordinary challenge to mental resilience. This study assesses mental health status during the COVID-19 pandemic and its associated factors among informal waste workers in Bangladesh. A cross-sectional survey was conducted in June 2020 among 176 informal waste workers selected from nine municipalities and one city corporation in Bangladesh. General Health Questionnaire (GHQ-12) was used to assess respondents’ mental health. The study found that 80.6% of the individuals were suffering from psychological distress; 67.6% reported anxiety and depression, 92.6% reported social dysfunction, and 19.9% reported loss of confidence. The likelihood of psychological distress (Risk ratio [RR]: 1.23, 95% confidence interval [CI]: 1.02–1.48) was significantly higher for female than male. Multiple COVID-19 symptoms of the family members (RR: 1.20, 95% CI: 1.03–1.41), unawareness about COVID-19 infected neighbor (RR: 1.21, 95% CI: 1.04–1.41), income reduction (RR: 1.60, 95% CI: 1.06–2.41) and daily household meal reduction (RR: 1.34; 95% CI: 1.03–1.73) were also found to be associated with psychological distress. These identified factors should be considered in policy-making and support programs for the informal waste workers to manage the pandemic situation as well as combating COVID-19 related psychological challenges.

## Introduction

The deadliest global pandemic, the coronavirus disease 2019 (COVID-19), started in December 2019 in Wuhan, China [1]. The virus spread worldwide so quickly that the World Health Organization (WHO) has declared the COVID-19 outbreak as a pandemic on March 2020. By the time of writing (25 November, 2021), more than 258 million confirmed COVID-19 cases and more than 5.17 million deaths have been reported globally [2]. To prevent the transmission of coronavirus, governments across the world have imposed restrictive measures, such as social distancing, voluntary isolation, or lockdowns [3–6]. People were ordered to stay-at-home, educational institutions were closed, and many workers were furloughed, laid off, or told to work from home. All of these have unsettled people’s lives, and have severe implications for health and wellbeing [7–9].

Global prompt expansion of COVID-19 presents unprecedented public health emergencies that affected the health, safety, and well-being of both individuals and communities. These effects may lead to a range of mental disorders in affected people as well as in general population [10,11]. However, adult people, people from the lower-income cluster, and urban areas have shown greater risk of developing mild to severe stress, anxiety, depression, and post-traumatic symptoms anxiety in COVID-19 [12]. The fear of uncertainty, specifically, financial uncertainty; severe shortages of food supply and resources for testing and treatment; having underlying diseases; family concerns; fear of infection; close contact with COVID-19 infected people; absence of physical exercise, and limited or no recreational activity during the outbreak of coronavirus [13–15] lead individuals to widespread depression, fear, panic, and anxiety [11,16].

Although individuals from all professions are contributing their best to fight against COVID-19, research documented that essential workers such as healthcare workers, social care and transport workers are at a greater risk of being affected by severe COVID-19 [17]. Sanitation and waste workers, an unfocused type of essential and critical front-line workers, are generally marginalized and live-in congested colonies, slums, or informal settlements with limited access to basic services that increase the risk of any infection, injury, and death especially in developing countries like Bangladesh. They have to collect waste daily from houses and public places, especially from hospitals which are highly vulnerable place for the virus to spread out. The nature of their working and living conditions make them vulnerable to come in contact of COVID-19 infected people. Moreover, they are usually exposed to a large population and have less possibility to maintain physical distance than other occupations. To protect human health during all infectious disease outbreaks including COVID-19, the provision of safe water, sanitation and waste management and hygiene conditions is crucial, and the sanitation and waste workers are providing such essential operational support during the COVID-19 pandemic [18]. Therefore, understanding mental health condition of sanitation and waste workers during the outbreak of COVID-19 is important to confront the pandemic faced by the world.

Bangladesh identified its first COVID-19 case on 8 March 2020 and as of 25 November 2021, the total confirmed cases are 1.57 million, of them, 11,796 died [2]. The situation possesses psychological impact on different group of population of the country with varying proportions [13,19–21]. For instance, Banna et. al. [12] reported that the prevalence of anxiety and depressive symptoms in adult population is 33.7% and 57.9%, respectively. Studies among students document poor mental health during the COVID-19 pandemic; the prevalence of depression ranges from 46.9% to 72.0%, and anxiety from 18.1% to 40.0% [13,21]. Children are also suffering from mild to severe mental health disturbances during the period of lockdown where the prevalence varied from 7.2% to 30.5% [20]. Essential front-line health workers such as physicians are also suffering from anxiety and depression with a higher rate during the pandemic [22,23]. However, little is known about the mental health condition of waste workers who are a vulnerable working group and essential workers during the outbreak of COVID-19. Approximately, 40,000 informal waste workers, who conduct waste recovery activities outside any official framework and are not acknowledged as an occupational group in the national database, are working in Bangladesh [24]. Their contributions is not legally accredited and acknowledged by the government or even by society, therefore, are normally excluded from the social security system and other benefits. They usually work without adequate protection and are at higher risk of getting infected by coronavirus [25]. This study aims to assess mental health condition during the COVID-19 pandemic and its associated factors among informal waste workers in Bangladesh.

## Data and methods

### Study areas and design

This study was a secondary analysis of data of a study ‘Immediate Impact of Coronavirus on Waste Workers in 10 City Corporation/Municipalities in Bangladesh’, which was a cross-sectional study conducted among informal waste workers in nine Municipalities under eight administrative districts namely Cumilla, Magura, Meherpur, Rajbari, Faridpur, Bagerhat, Barguna, Satkhira and a City Corporation in Gazipur district in Bangladesh. These areas and targeted waste workers are covered under a project “Dignifying Lives: inclusive approach for socioeconomic empowerment of informal waste and sanitation workers” implemented by Practical Action (an International Charity Organization) to improve waste workers’ wellbeing and occupational safety. Waste workers include pit emptier, waste collector, inorganic trader, and cleaners/sweepers. In these areas the waste workers have a total of 46 cooperatives, and the waste workers belong to these cooperatives were eligible to participate. Data collection for this cross-sectional study was done in June 2020.

### Sample size determination and sampling

A stratified random sampling approach was used to select the respondents. We used a single population proportion formula for known population (N = 1795) to determine the required sample based on the following assumption: proportion of population with certain characteristics, *p* = 0.5, precision level (margin of error) of 7%, and a confidence level of 95%. This accuracy rate or amount of admissible marginal error was chosen due to hard to reach to the respondents during countrywide lockdown and mobility restriction. Samples were distributed proportionately to each of the cooperatives. Respondents from these cooperatives were then selected randomly; at least three respondents were selected from each of the 46 cooperatives. Information on background and socioeconomic variables, COVID-19 related characteristics, and mental well-being were collected from 176 informal waste workers.

### Data collection and quality assurance

Interviews were conducted using a well-structured questionnaire. Maintaining the COVID-19 preventive guidelines of WHO [26], data collection were conducted using a face-to-face interview. However, due to strict restriction in mobility and lockdown imposed by the Government of Bangladesh all over the country, interview of 106 respondents (60%) were conducted over mobile phone. The survey tools were pretested before finalizing. Interviews were conducted by well-trained interviewers and they further verified and cross-checked the collected data to minimize the data collection errors. Additional details of data collection are available elsewhere [27]. This study was carried out in accordance with the Declaration of Helsinki as revised in 2013. Informed consent was obtained from all participants. The project study has got approval from the Non-governmental Affairs Bureau (NGOAB) of Bangladesh.

### Outcome variables

Mental health status, quantified by the magnitude of psychological distress, of the waste workers during the COVID-19 outbreak was the outcome of interest in this current analysis. General Health Questionnaire (GHQ-12), a self-reported 12-item scale, was used to measure psychological distress among the respondents. The GHQ-12 consists of an equal number of positive and negative items that asked respondents to indicate their agreement on a four-point scale ranging from not at all (= 0) to more than usual (= 3) across items. The total score of the GHQ-12 can range from 0 to 36, with a higher score suggesting higher psychological distress. Besides unidimensional model, we further used the 3-factor model where the factors are anxiety and depression (4 items), social dysfunction (6 items), and loss of confidence (2 items) [28]. Based on earlier documentation [29,30], the presence of any poor mental health conditions was measured by using the median point thresholds. The GHQ-12 is widely validated in both developed and developing countries including Bangladesh [29,31–33]. All items are available in “S1 Table”.

### Explanatory variables

Guided by existing literature, a set of demographics, economic and health-related factors were examined in this study as explanatory variables. A complete list of explanatory variables, their categories and coding are presented in Table 1.

**Table 1:** Details of independent variables.

Variables	Asked questions	Answers	Coding
Age	Please mention about your age	Collected as continuous format in years	1 = ≤ 25 year; 2 = 26–50 years; 3 = 50 < years
Gender	Please mention about gender		1 = Male; 2 = Female
Educational status	How many formal educational years you have completed?	Collected as continuous format in years, then further recoded in three categories	0 = No education; 1 = Up to primary; 2 = Up to secondary or higher
Occupation type	What is your type of occupation?	1 = Pit emptier; 2 = Waste collector; 3 = Inorganic trader; 4 = Cleaners/ sweeper	
Earing member in the family	How many earning members you have in your household?	Collected in no. of earning members in family	
Monthly household income (in BDT)	What is the monthly income of your household?	Collected in BDT for during COVID-19 and before COVID-19	
Symptoms related to COVID-19 faced by respondents ^a^	What COVID-19 related symptoms you faced during pandemic?	Respondents answered several i.e., nothing faced yet, fever, cough, difficulty in breathing, sore throat, sneezing (include runny nose, cold feeling), fatigue (tiredness), aches and pains (mostly muscle and chest), headaches, diarrhea, poor appetite and vomiting	0 = No symptoms 1 = Single symptom 2 = Multiple symptoms
Symptoms related to COVID-19 faced by respondents’ family members ^a^	What COVID-19 related symptoms your family members faced during pandemic?		
Had any chronic diseases	Do you have any chronic diseases?	Answered taken for no chronic diseases, hypertension, heart disease, diabetes, respiratory diseases (Chronic obstructive pulmonary disease-COPD, asthma, etc.), kidney disease, and liver problem.	0 = No 1 = Yes
Had COVID-19 infected neighbor	Do you have any COVID-19 infected neighbor in your community?	0 = No; 1 = Yes; 2 = Don’t know	
Arranged food from savings	Do you arrange food for family from savings?		0 = No; 1 = Yes
Arranged food from borrowing	Do you arrange food for family by borrowing?		0 = No; 1 = Yes
Income reduced	Calculated from during COVID-19 and before COVID-19 income of the households		0 = No; 1 = Yes
Daily meal reduced	How many times your family are taking meal sufficiently?	Collected and calculated from during COVID-19 and before COVID-19 income of the households	0 = No; 1 = Yes

Note:

^a^In last 1 month prior to survey.

### Statistical analysis

The prevalence of psychological distress, anxiety and depression, social dysfunction, and loss of confidence was assessed for the study population. Assessment of the extent to which different factors represents a marker for waste workers’ overall psychological distress, anxiety and depression, social dysfunction, and loss of confidence during the COVID-19 pandemic was conducted by using a generalized estimating equation-modified Poisson regression approach with robust error variance option to produce a direct assessment of risk ratios (RRs) [34]. Using this approach adjusted models were fitted for different binary outcome variables with different factors as predictors. The risk ratios were estimated to measure the strength of association, and a 95% confidence interval (CI) was computed for significance testing. In all analyses, the significance was set at *p*<0.05. Data were analyzed using computer program STATA windows version 14.0 SE (StataCorp. LP, College Station, TX, USA).

## Results

### Background characteristics

Table 2 represents the socio-economic and demographic profile of the study population. Most of the surveyed individuals were aged 26–50 years (75.6%) and were male (55.0%). Nearly, one-third (33.5%) of the individuals had no formal education, while 39.2% of them were educated up to primary-level. Of these surveyed individuals, 38.6% were cleaner/sweepers, 28.4% were waste collectors, 17.6% were pit emptier and 15.3% were inorganic traders. Before the COVID-19 pandemic, these individuals had an average of 1.74 (SD: ± 0.78) earning members in the family and that reduced to 1.43 (SD: ± 0.71) during the COVID-19 period. The mean household income of these respondents was also reduced by 5469.03 (SD: ± 4040.58) Taka (before the COVID-19 pandemic, mean ± SD: 13322.21 ± 5418.61; and during the COVID-19 pandemic, mean ± SD: 7853.01 ± 4446.00) (1 USD = 85.0 Taka).

**Table 2:** Socio-economic and demographic profile of study population.

**Characteristics**	**n**	**%**
**Age**		
≤ 25 years	23	13.1
26–50 years	133	75.6
50 < years	20	11.4
**Gender**		
Male	97	55.0
Female	79	45.0
**Educational status**		
No education	59	33.5
Up to primary	69	39.2
Up to secondary or higher	48	27.3
**Occupation types**		
Pit emptier	31	17.6
Waste collector	50	28.4
Inorganic trader	27	15.3
Cleaners/ sweeper	68	38.6
**Economic information**	**Mean**	**± SD**
**Earing member in the family**		
Before COVID-19	1.74	± 0.78
During COVID-19	1.43	± 0.71
Reduction (before vs during COVID-19)	0.32	± 0.59
**Monthly household income (in BDT)**		
Before COVID-19	13322.21	± 5418.61
During COVID-19	7853.01	± 4446.00
Reduction (before vs during COVID-19)	5469.03	± 4040.58

### COVID-19 symptoms and mental health condition

As displayed in Table 3, 48.9% of the respondents had experienced COVID-19 related symptoms during the pandemic that includes fever (22.2%), cough (16.5%), aches and muscle pains (11.4%), and sneezing (10.2%). In addition, around 43% of the respondents’ family members also had COVID-19 symptoms. However, only 56.3% of these respondents had approached to any treatment facilities but no one called the hotline number that particularly assigned by Institute of Epidemiology, Disease Control and Research (IEDCR), Bangladesh to ensure treatment for COVID-19. About 27.0% of the respondents had reported to have a chronic disease like hypertension, diabetes, or heart diseases.

**Table 3:** COVID-19 related symptoms, approach to treatment faced by the waste workers.

Characteristics	Experienced by respondents		Experienced by respondents’ family members’	
	n	%	n	%
**Symptoms related to COVID-19**				
No symptoms	90	51.1	100	56.8
Single symptom	34	19.3	33	18.8
Multiple symptoms	52	29.6	43	24.4
**Types of symptoms** ^1^				
Fever	39	22.2	46	26.1
Cough	29	16.5	28	15.9
Difficulty in breathing	11	6.3	8	4.6
Sore throat	9	5.1	5	2.8
Sneezing (include runny nose, cold feeling)	18	10.2	10	5.7
Fatigue (tiredness)	13	7.4	3	1.7
Aches and pains (mostly muscle and chest)	20	11.4	15	8.5
Headaches	17	9.7	15	8.5
Diarrhea	5	2.8	7	4.0
Poor appetite	3	1.7	1	0.6
Vomiting	8	4.6	2	1.1
**Approached to treatment** ^2^				
No	77	43.8	-	-
Yes	99	56.3	-	-
**Treatment place** ^1^			-	-
Not approached	77	43.8	-	-
Called hotline	0	0.0	-	-
Dispensary	53	54.0	-	-
Private clinic/doctor	26	26.0	-	-
Hospital	20	20.0	-	-
**Had any chronic diseases** ^3^			-	-
No	48	27.0	-	-
Yes	128	73.0	-	-

Note:

^1^ Multiple responses;

^2^For both individuals and their family members;

^3^Only for respondents and these diseases includes hypertension, diabetes, heart diseases, kidney diseases, respiratory diseases (Asthma, COPD, etc.), and liver problem.

Approximately four out of every five respondents (80.6%) were suffering from psychological distress (mean ± SD: 15.6 **±** 3.4); 67.6% reported anxiety and depression (mean ± SD: 6.1 **±**1.5), 92.6% reported social dysfunction (mean ± SD: 5.8 **±**3.4), and 19.9% reported loss of confidence (mean ± SD: 3.8 **±**1.4) (Table 4).

**Table 4:** Mental health status of waste workers during COVID-19.

Mental health status	Mean (± SD)	n	%
**Overall psychological distress** ^a^	15.6 (**±**3.4)		
No		33	18.8
Yes		142	80.6
**Anxiety and depression** ^b^	6.1 (**±**1.5)		
No		54	30.7
Yes		119	67.6
**Social dysfunction** ^c^	5.8 (**±**3.4)		
No		10	5.7
Yes		163	92.6
**Loss of confidence** ^d^	3.8 (**±**1.4)		
No		138	78.4
Yes		35	19.9

Note:

^a^ Score out of 36;

^b^ Score out of 12;

^c^ Score out of 18; and

^d^ Score out of 6.

### Associated factors of poor mental health conditions

Table 5 illustrates the associated factors of psychological distress, anxiety and depression, social dysfunction, and loss of confidence in this population. The study revealed that the likelihood of psychological distress (RR: 1.23, 95% CI: 1.02–1.48), social dysfunction (RR: 1.10, 95% CI: 1.01–1.20), and loss of confidence (RR: 1.67, 95% CI: 1.07–2.59) was significantly higher for female than male, while female waste workers were 0.73 times (95% CI: 0.53–0.99) less likely to suffer from anxiety and depression. Respondents with up to primary-level education were 1.91 times (95% CI: 1.17–3.29) more likely to experience loss of confidence during the pandemic compared to individuals with no education. Experiencing multiple COVID-19 symptoms by individuals had 1.11 times (95% CI: 1.02–1.21) higher likelihood of developing social dysfunction than individuals who had not experienced any such symptoms. Compared to the respondents whose family members did not experience any COVID-19 symptoms, respondents whose family member experienced multiple COVID-19 symptoms were more likely to report psychological distress (RR: 1.20, 95% CI: 1.03–1.41), and anxiety and depression (RR = 1.89,95% CI: 1.39–2.57). The waste workers who had pre-existing chronic disease were less likely to experience social dysfunction (RR: 0.87 95% CI: 0.79–0.96). Respondents who did not know whether he had any COVID-19 infected neighbors or not were more likely to develop psychological distress (RR: 1.21, 95% CI: 1.04–1.41) and social dysfunction (RR: 1.07, 95% CI: 1.00–1.13) than who knew that he had no COVID-19 infected neighbors. Psychological distress was also higher among respondents whose income (RR: 1.60, 95% CI: 1.06–2.41) and daily household meal (RR: 1.34; 95% CI: 1.03–1.73) were reduced during the COVID-19 outbreak.

**Table 5:** Association of psychological distress, anxiety and depression, social dysfunction and loss of confidence with possible influencing factors in surveyed nine municipalities and one city-corporation during COVID-19 in Bangladesh.

Measures	Overall psychological distress	Anxiety and depression	Social dysfunction	Loss of confidence
	Adjusted RR (95% CI)	Adjusted RR (95% CI)	Adjusted RR (95% CI)	Adjusted RR (95% CI)
**Age**				
≤ 25 years	1.00	1.00	1.00	1.00
26–50 years	0.91 (0.68–1.21)	0.86 (0.61–1.20)	1.01 (0.85–1.19)	0.85 (0.51–1.41)
50 < years	0.88 (0.62–1.25)	0.84 (0.50–1.42)	0.97 (0.80–1.19)	0.53 (0.19–1.43)
**Gender**				
Male	1.00	1.00	1.00	1.00
Female	1.23 (1.02–1.48) *	0.73 (0.53–0.99) *	1.10 (1.01–1.20) *	1.67 (1.07–2.59) *
**Educational status**				
No education	1.00	1.00	1.00	1.00
Up to primary	0.97 (0.79–1.17)	1.24 (0.85–1.81)	0.98 (0.91–1.17)	1.91 (1.17–3.29) *
Up to secondary or higher	0.87 (0.67–1.14)	1.33 (0.87–2.03)	0.94 (0.84–1.06)	1.51 (0.76–2.96)
Occupation types				
Pit emptier	1.00	1.00	1.00	1.00
Waste collector	0.95 (0.77–1.16)	1.01 (0.67–1.49)	1.03 (0.92–1.16)	0.54 (0.31–0.93) *
Inorganic trader	0.86 (0.66–1.12)	1.22 (0.80–1.87)	1.01 (0.87–1.17)	0.60 (0.30–1.20)
Cleaners/ sweeper	0.76 (0.59–0.97) *	1.40 (0.90–2.20)	0.99 (0.89–1.12)	0.45 (0.28–0.74) **
**Symptoms related to COVID-19 experienced by respondents**				
No symptoms	1.00	1.00	1.00	1.00
Single symptoms	0.96 (0.74–1.23)	0.93 (0.67–1.30)	1.06 (0.96–1.17)	0.98 (0.57–1.71)
Multiple symptoms	1.06 (0.89–1.26)	0.86 (0.64–1.16)	1.11 (1.02–1.21) **	1.07 (0.66–1.73)
**Symptoms related to COVID-19 experienced by respondents any family members**				
No symptoms	1.00	1.00	1.00	1.00
Single symptoms	0.84 (0.61–1.15)	1.65 (1.19–2.28) **	1.04 (0.91–1.18)	0.68 (0.36–1.28)
Multiple symptoms	1.20 (1.03–1.41) *	1.89 (1.39–2.57) ***	1.06 (0.98–1.14)	1.01 (0.63–1.64)
**Had any chronic diseases**				
No	1.00	1.00	1.00	1.00
Yes	0.93 (0.77–1.11)	1.05 (0.78–1.42)	0.87 (0.79–0.96) **	1.21 (0.76–1.92)
**Had COVID-19 infected neighbor**				
No	1.00	1.00	1.00	1.00
Yes	0.68 (0.45–1.03)	0.67 (0.41–1.09)	0.98 (0.85–1.12)	1.38 (0.68–2.79)
Don’t know	1.21 (1.04–1.41) **	1.01 (0.72–1.39)	1.07 (1.00–1.13) *	1.35 (0.88–2.07)
**Arranged food from savings**				
No	1.00	1.00	1.00	1.00
Yes	0.92 (0.71–1.21)	1.09 (0.75–1.57)	0.92 (0.78–1.09)	0.66 (0.35–1.27)
**Arranged food from borrowing**				
No	1.00	1.00	1.00	1.00
Yes	0.90 (0.75–1.08)	1.12 (0.85–1.47)	1.02 (0.93–1.13)	0.96 (0.64–1.43)
**Income reduced**				
No	1.00	1.00	1.00	1.00
Yes	1.60 (1.06–2.41) *	0.84 (0.59–1.21)	1.15 (0.95–1.39)	0.84 (0.51–1.34)
**Daily meal reduced**				
No	1.00	1.00	1.00	1.00
Yes	1.34 (1.03–1.73) *	1.06 (0.79–1.43)	1.02 (0.92–1.14)	1.12 (0.71–1.76)

## Discussion

To our knowledge, this is the first study that assesses the mental health status and identifies the factors associated with developing poor mental health among informal waste workers during the COVID-19 pandemic in Bangladesh. This study found that 81% waste workers have experienced psychological distress during the COVID-19 outbreak. Specifically, 93% reported social dysfunction, 67% reported anxiety and depression, and 20% of waste workers reported loss of confidence. These findings are consistent with the findings of prior studies from other countries as well as Bangladesh for other population cluster [12–15]. We also found that around 50% of the waste workers and their family members had experienced any symptoms of COVID-19 (mostly fever and cough), which is similar to the general population [35–37]. Moreover, during the outbreak of COVID-19, both earning members in the family and income of the waste workers’ reduced. The outbreak had a major negative impact on the global economy and employment, and people lost their jobs as a result of the outbreak and related movement restriction or preventive measures [38].

This study revealed a rate of psychological disorders among informal waste workers which is much higher than that is reported among front-line health workers during the COVID-19 pandemic in some global studies [14,39–41]. The difference in the prevalence of poor mental health status between essential front-line waste workers and health workers could be explained in several ways. For example, collecting waste is a type of informal employment and a study in Brazil has documented that the levels of mental disorders are found to be higher among informal workers relative to formally employed workers [42]. The poorest and most vulnerable people, like homeless, elderly, women, children, chemical dependents, and ethnic minority people generally engage in this informal job because of unemployment, poverty or out of desperation, which is considered as a ‘poor quality’ job [43,44]. Research revealed that having a ‘poor quality’ or hated job might contribute to poor mental health [45]. Moreover, informal waste workers often live in isolation and are subject to social stigma, bullying, and conflict as well as low self-esteem- all of which may negatively affect their psychological wellbeing [43,46]. Another explanation of higher rate of poor mental health among informal waste workers could be precarious working conditions with insufficient protections against occupational hazards associated with their work, such as having poor access to and use of formal personal protective equipment (PPE) [47]. Along with this generalized concern about unprotected working conditions, fear of COVID-19 infection, lack of knowledge and awareness about the infection, low earnings, financial insecurity, nature of the job might make these informal waste workers more vulnerable to mental health issues than other front-line health workers [48]. Therefore, higher mental health issues that informal waste workers encountered during this pandemic reported in this study will help policy makers to develop intervention strategies for improving mental health of this vulnerable group.

This study identified several factors that are associated with poor mental health of the waste workers. We found that females are more likely to suffer from psychological distress, specifically social dysfunction and loss of confidence than males. Global findings suggest that the risk of poor mental health is usually much higher in women due to lack of adequate domestic and emotional support [49,50]. The pandemic has affected women severely than men both at workplace and at home by increasing workload and violence against women during lockdown and quarantine measures [49]. A recent systematic review suggests that to prevent COVID-19 infection, early detection and prompt treatment is urgent, where proper social and familial support is a key protective factor [51].

We also found that waste workers with multiple COVID-19 related symptoms were more likely to report social dysfunction. However, if any family member of the respondents shows multiple symptoms of COVID-19, the respondents were more likely to report psychological distress, specifically anxiety and depression. People usually do care for their family more than their own; thus, illness or symptoms related to COVID-19 of any family members have a direct negative consequence on their own mental health [52,53]. Poor mental health during the COVID-19 outbreak could be due to experiencing uncertainty and becoming very concerned about the symptoms resembling COVID-19 infection. Understanding the potential threat and impact of COVID-19 infection, they may become more worried and they do not have enough health information to protect them or their family members-all of which increase psychological distress [54].

We observed that the waste workers who didn’t know whether they have COVID-19 infected person in their neighborhood or not, were more likely to suffer from psychological distress and social dysfunction symptoms. When an individual doesn’t know exactly whether they have infected neighbors or not, that increases the fear of illness and severe uncertainty which contributes to widespread psychological distress and behavioral disorders [11]. This kind of uncertain situation always causes more damage mentally than the virus itself [55,56].

The COVID-19 pandemic and the resulting isolation, job loss and economic recession might have negative effect on people’s mental health. In support of this, we found that income reduction increased the risk of developing poor mental health symptoms in waste workers. This is in line with other existing literature [57,58]. Usually, income loss and financial strain are uniquely associated with poor mental health i.e., anxiety and depressive symptoms [58–60]. Many people lost their jobs and income due to lockdown, and had to borrow loan from others in the current pandemic situation, which ultimately ended up stressing them out [57,59] and even that exacerbates poor mental health symptoms over time, above and beyond pandemic-related mental illness [58]. Moreover, in this pandemic situation, many households of these waste workers did not have enough food and severely faced food crisis which ultimately created mental pressure on them and might develop poor mental health symptoms. Food insecurity is directly linked with poor mental health and a specific mental stressors worldwide [61]. Researchers repeatedly found that economic hardship and food crisis are interrelated and that causes potential mental stress of mass people [60,62,63]. Even people have attempted suicide after failing in coping up with economic loss and food insecurity during the COVID-19 pandemic [64]. Thus, we suggest the need for more specific strategies to reduce mental health disorders in a pandemic situation.

### Limitations and strengths

To interpret our study results, several limitations should be kept in mind. First, this study was only conducted in nine municipalities and a city corporation, and therefore, we cannot generalize the results to the whole country. Second, the cross-sectional design of this study limited our ability to identify causal relationships between mental health status and its possible risk factors during the COVID-19 period. Third, information before the COVID-19 outbreak can potentially cause substantial recall bias. Fourth, the waste workers were asked to self-report their experience of mental health related questions; therefore, there could be a possibility of under or over reporting of mental health status. Fifth, during the COVID-19 pandemic, daily working hours of the front-line workers appeared as one of the important risk factors for psychological disorders, including depression, anxiety, somatization, insomnia and suicide risk [39]. We were unable to include this important risk factor in our analyses due to utilization of secondary data. Finally, the GHQ-12 is a widely used screening instrument to measure of mental health outcomes, but it is not a diagnostic tool. Limitations notwithstanding, this study has the potential to support the development and/or modification of prevention strategies for the COVID-19 pandemic. We are unaware of any previous studies that have assessed the mental health status of this marginalized vulnerable group of population. Therefore, this study could be the first to have reported mental health status of informal waste workers and its associated factors in Bangladesh.

### Conclusion

This study found that a very higher proportion of informal waste workers suffered from psychological distress during the COVID-19 pandemic. Factors, such as experiencing multiple COVID-19 symptoms by individuals and their family members, income reduction, daily household meal reduction, are identified as contributing factors for poor mental health among the waste workers. Designing of intervention programs aimed to improve mental health status of the informal waste workers during emergencies should consider these factors. We expect that the government pay particular attention to these disadvantaged but essential front-line workers for their needs during the pandemic and formulate a time-oriented policy and support to improve their mental health as well as combating COVID-19 related psychological challenges.

## Supporting information

S1 Table Details of GHQ-12 tool. (PDF)
