# Supplementary material for: Mental health status of informal waste workers during the COVID-19 pandemic in Bangladesh
Source: PLoS One. 2022 Jan 7;17(1):e0262141. doi: 10.1371/journal.pone.0262141 (PMC8741044; doi:10.1371/journal.pone.0262141)
Supplement: S1 Table — (PDF) [file pone.0262141.s001.pdf]

S1 Table. Details of the GHQ-12 tool.

**Over all Psychological well-being**	
Social dysfunction	
• Have you recently been able to, concentrate on what you are doing?	0 = never, 1 = sometimes, 2 = often, and 3 = always
• Have you recently felt you were playing important part in things?	0 = never, 1 = sometimes, 2 = often, and 3 = always
• Have you recently been feeling reasonably happy, all things considered?	0 = never, 1 = sometimes, 2 = often, and 3 = always
• Have you recently felt capable of making decisions about things?	0 = never, 1 = sometimes, 2 = often, and 3 = always
• Have you recently been able to enjoy your normal day to day activity?	0 = never, 1 = sometimes, 2 = often, and 3 = always
• Have you recently been able to face up to your problems?	0 = never, 1 = sometimes, 2 = often, and 3 = always
Anxiety and depression	
• Have you recently felt consistently under strain?	0 = never, 1 = sometimes, 2 = often, and 3 = always
• Have you recently felt you couldn’t overcome your difficulties?	0 = never, 1 = sometimes, 2 = often, and 3 = always
• Have you recently lost much sleep over worry?	0 = never, 1 = sometimes, 2 = often, and 3 = always
• Have you recently been unhappy and depressed?	0 = never, 1 = sometimes, 2 = often, and 3 = always
Loss of confidence	
• Have you recently been losing confidence in yourself?	0 = never, 1 = sometimes, 2 = often, and 3 = always
• Have you recently been thinking of yourself as a worthless person?	0 = never, 1 = sometimes, 2 = often, and 3 = always
